# The Significance of Cell Surface N-Glycosylation for Internalization and Potency of Cytotoxic Conjugates Targeting Receptor Tyrosine Kinases

**DOI:** 10.3390/ijms23158514

**Published:** 2022-07-31

**Authors:** Marta Poźniak, Dominika Żukowska, Aleksandra Gędaj, Mateusz Adam Krzyścik, Natalia Porębska, Małgorzata Zakrzewska, Jacek Otlewski, Łukasz Opaliński

**Affiliations:** Faculty of Biotechnology, Department of Protein Engineering, University of Wroclaw, Joliot-Curie 14a, 50-383 Wroclaw, Poland; marta.latko2@uwr.edu.pl (M.P.); dominika.zukowska@uwr.edu.pl (D.Ż.); aleksandra.matynia2@uwr.edu.pl (A.G.); mateusz.krzyscik@uwr.edu.pl (M.A.K.); natalia.porebska2@uwr.edu.pl (N.P.); malgorzata.zakrzewska@uwr.edu.pl (M.Z.); jacek.otlewski@uwr.edu.pl (J.O.)

**Keywords:** N-glycosylation, endocytosis, galectins, cytotoxic conjugates, cancer therapy, RTK

## Abstract

Precise anticancer therapies employing cytotoxic conjugates constitute a side-effect-limited, highly attractive alternative to commonly used cancer treatment modalities, such as conventional chemotherapy, radiotherapy or surgical interventions. Receptor tyrosine kinases are a large family of N-glycoproteins intensively studied as molecular targets for cytotoxic conjugates in various cancers. At the cell surface, these receptors are embedded in a dense carbohydrate layer formed by numerous plasma membrane glycoproteins. The complexity of the cell surface architecture is further increased by galectins, secreted lectins capable of recognizing and clustering glycoconjugates, affecting their motility and activity. Cell surface N-glycosylation is intensively remodeled by cancer cells; however, the contribution of this phenomenon to the efficiency of treatment with cytotoxic conjugates is largely unknown. Here, we evaluated the significance of N-glycosylation for the internalization and toxicity of conjugates targeting two model receptor tyrosine kinases strongly implicated in cancer: HER2 and FGFR1. We employed three conjugates of distinct molecular architecture and specificity: Affibody_HER2_-vcMMAE (targeting HER2), vcMMAE-KCK-FGF1.E and T-Fc-vcMMAE (recognizing different epitopes within FGFR1). We demonstrated that inhibition of N-glycosylation reduced the cellular uptake of all conjugates tested and provided evidence for a role of the galectin network in conjugate internalization. In vitro binding studies revealed that the reduced uptake of conjugates is not due to impaired HER2 and FGFR1 binding. Importantly, we demonstrated that alteration of N-glycosylation can affect the cytotoxic potential of conjugates. Our data implicate a key role for cell surface N-glycosylation in the delivery of cytotoxic conjugates into cancer cells.

## 1. Introduction

Cytotoxic conjugates (CCs), mainly in the form of antibody–drug conjugates (ADCs), are a highly effective weapon against various cancers [1]. Several ADCs have been already approved for cancer treatment, and their prominence in oncology is rapidly increasing, with numerous ADCs currently under clinical trials [2]. The major advantage of CCs over conventional anticancer chemotherapy is their precision. These potent cytotoxic agents selectively recognize cancer cells, leading to their death, and largely omit healthy cells, thus limiting the undesirable side effects of therapy [1]. CCs are composed of three major components: a targeting molecule (usually a monoclonal antibody, alternatively a peptide or receptor ligand) and a highly potent cytotoxic drug (too toxic for untargeted usage) linked together by a peptide linker [1,2]. The precision of CC action is ensured by a targeting molecule, which recognizes cancer-specific cell surface receptor proteins (targets) and enables intracellular delivery of cytotoxic drugs via receptor-mediated endocytosis [3].

Ideal targets for CCs are proteins that are expressed exclusively on the surface of cancer cells and exhibit high endocytic potential [4]. Receptor tyrosine kinases (RTKs) are plasma membrane N-linked glycoproteins that transmit signals from the extracellular environment to the cell interior. RTK signaling governs basic cellular processes such as division, differentiation, motility, metabolism and death, regulating the development and homeostasis of the human body. In many cancers, RTKs are overexpressed, facilitating proliferation, survival and spreading of cancer cells; therefore, these receptors are intensively explored targets for CCs [5]. RTKs, along with other plasma membrane and secreted glycoproteins, form a dense glyco-layer on the surface of cancer cells that regulates cell physiology and modulates cell access to external molecules [6]. Altered RTK glycosylation is observed during oncogenesis, where it facilitates tumor cell proliferation, survival and metastasis through various mechanisms, including modulation of RTK activity and receptor endocytosis [7]. Furthermore, N-glycosylation of RTKs promotes interaction with galectins, a family of secreted lectins strongly implicated in cancer, which can either directly activate RTKs or lead to sustained RTK signaling by manipulating RTK endocytosis [6,8,9].

In the dense network of cell surface glycoconjugates, CCs must precisely recognize cancer-specific RTKs and ensure receptor-mediated endocytosis of the conjugates, delivering the toxic drug inside a cancer cell, leading to cell death [1]. Modifications of cell surface glycosylation may represent an important but as yet unexplored factor, influencing the efficacy of targeted anticancer therapies. Therefore, in this study, we decided to assess the significance of cell surface N-glycosylation for the uptake and potency of CCs targeting model RTKs.

## 2. Results

### 2.1. The Contribution of Cell Surface N-Glycosylation to the Uptake of CCs Targeting RTKs

**Figure 1:**
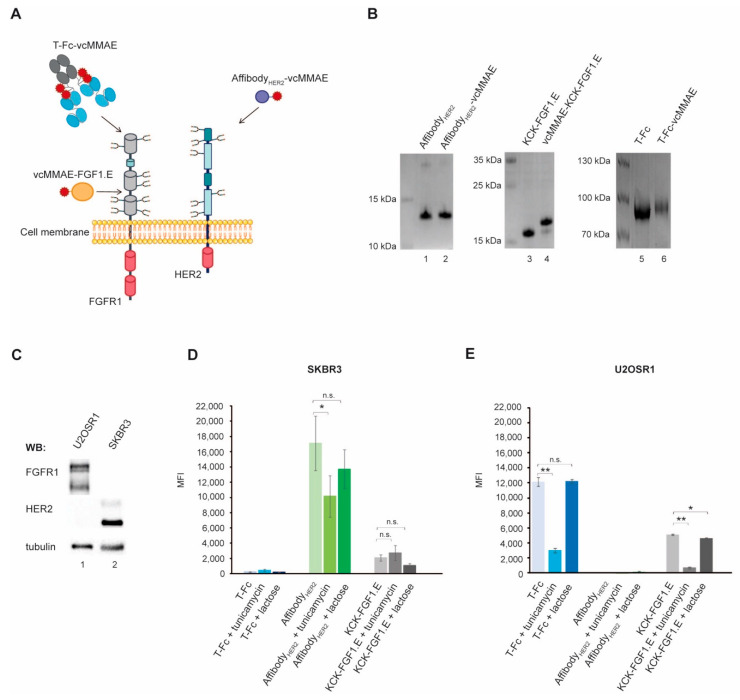
The effect of N-glycosylation and galectins on the internalization of CCs. (**A**) Hypothetical model of FGFR1 and HER2 domain organization and membrane topology with putative N-glycosylation sites marked. The scheme of cytotoxic conjugates targeting FGFR1: vcMMAE-FGF1.E and T-Fc-vcMMAE and HER2: Affibody_HER2_-vcMMAE are shown. (**B**) The efficiency of vcMMAE conjugation to Affibody_HER2_, KCK-FGF1.E and T-Fc and purity of targeting molecules and conjugates Affibody_HER2_-vcMMAE, vcMMAE-KCK-FGF1.E and T-Fc-vcMMAE analyzed by SDS-PAGE. (**C**) FGFR1 and HER2 expression levels in the studied cell lines were analyzed by Western blotting using anti-FGFR1 and anti-HER2 antibodies. Tubulin level assessed with anti-tubulin antibody served as a loading control. (**D**,**E**) The efficiency and selectivity of T-Fc, Affibody_HER2_ and KCK-FGF1.E internalization under different conditions were studied by flow cytometry. Internalization was analyzed in serum-starved SKBR3 (**D**) and U2OSR1 cells (**E**). Cells were incubated with tunicamycin 24 h before the experiment or lactose 15 min before the experiment. Then cells were treated with T-Fc or Affibody_HER2_ or KCK-FGF1.E labeled with DyLight550. After 30 min incubation on ice, cells were transferred to 37 °C for 20 min, the cell surface was extensively washed to remove cell-bound, non-internalized proteins, and then cells were subsequently analyzed by flow cytometry. Results presented are mean values of three experiments ± SEM. The *t*-test was used to assess the statistical significance of measured differences in internalization; * *p* < 0.05, ** *p* < 0.01, n.s.—not significant.

In this study, we focused on two model RTKs that are overexpressed by several tumors: human epidermal growth factor receptor 2 (HER2) and fibroblast growth factor receptor 1 (FGFR1) [10,11]. These RTKs are N-linked glycoproteins, with HER2 containing seven and FGFR1 eight putative N-glycosylation sites [9,12]. We employed three CCs with different specificities and molecular architectures that demonstrated high selectivity toward their targets: (1) Affibody_HER2_-vcMMAE, consisting of HER2-specific three-helix engineered protein ZHER2:2891 derived from *Staphylococcal* protein A, conjugated to a valine–citrulline linker bearing monomethyl auristatin E (vcMMAE), highly specific for HER2, (2) vcMMAE-KCK-FGF1.E, a conjugate based on fibroblast growth factor 1 (FGF1) cysteine-free mutant with three additional stabilizing substitutions and an N-terminal KCKSGG linker (facilitating site-specific conjugation with vcMMAE), recognizing the D2 and D3 domains of FGFR1; (3) T-Fc-vcMAME, a tetravalent conjugate with superior endocytic potential constructed with engineered antibody fragments recognizing the D1 domain of FGFR1 (Figure 1A) [13,14,15,16]. We efficiently produced and purified targeting molecules for all studied CCs—Affibody_HER2_, KCK-FGF1.E and T-Fc (Figure 1B, lanes 1, 3, 5)—and effectively conjugated these targeting molecules with vcMMAE in a site-specific manner, yielding highly pure CCs (Figure 1B, lanes 2, 4, 6).

To study the significance of cell surface N-glycosylation for the internalization of CCs, we used two model cell lines: SKBR3 (HER2+, FGFR1-) and USOSR1 (HER2-, FGFR1+) (Figure 1C). Cells were treated with tunicamycin (a potent inhibitor of N-linked glycosylation) prior to incubation with fluorescently labeled targeting molecules: Affibody_HER2_, KCK-FGF1.E and T-Fc. We chose a concentration of tunicamycin that efficiently blocks cellular glycosylation while remaining largely neutral to the viability of the tested cells. Flow cytometry experiments revealed that treatment of SKBR3 and U2OSR1 cells with tunicamycin significantly inhibited the internalization of all studied targeting molecules (Figure 1D,E). At the same time, we observed no internalization of KCK-FGF1.E nor T-Fc into SKBR3 cells and Affibody_HER2_ into U2OSR1 cells, confirming the high specificity of the targeting molecules employed (Figure 1D,E). These data indicate that cell surface N-glycosylation is essential for effective receptor-mediated, selective internalization of CCs targeting HER2 and FGFR1.

### 2.2. Role of the Galectin Network in the Internalization of Conjugates

Information stored within N-glycans of RTKs can be read and converted into specific biological activities by galectins, a family of extracellular and intracellular lectins [9]. Galectins are also well-known endocytic mediators that facilitate the internalization of several N-glycosylated receptors, including RTKs [9,17]. To assess the involvement of galectins in the uptake of HER2- and FGFR1-specific CCs, cells were washed with lactose that outcompetes galectin binding to N-linked glycoconjugates prior to flow cytometry analyses. Lactose treatment had no significant effect on Affibody_HER2_ and T-Fc endocytosis efficiency but partially blocked KCK-FGF1.E uptake (Figure 1D). These data indicate a role for the galectin network in the endocytosis of KCK-FGF1.E–FGFR1 complexes.

**Figure 2:**
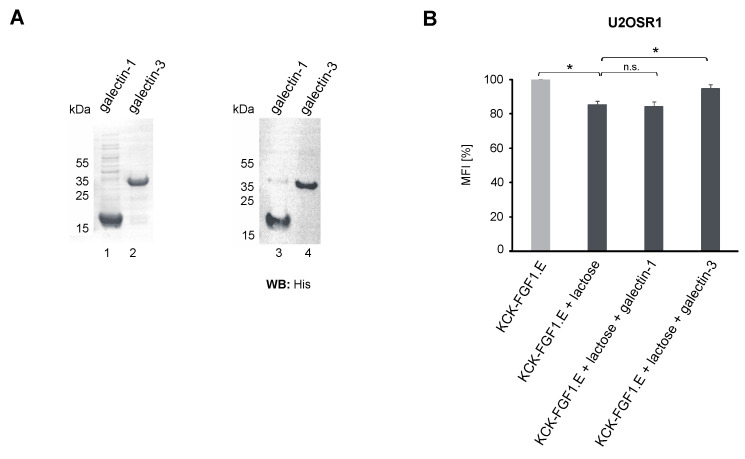
The impact of galectin-1 and -3 on the FGFR1-mediated uptake of KCK-FGF1.E. (**A**) Purity of recombinant galectin-1 and galectin-3 determined by SDS-PAGE (CBB) (**left panel**) and Western blotting using anti-His antibody (**right panel**). (**B**) Efficiency of KCK-FGF1.E internalization into U2OSR1 cells upon administration of recombinant galectins. Cells were treated with lactose for 15 min and then treated with KCK-FGF1.E labeled with DyLight550 in the presence of galectin-1 or galectin-3. After 30 min incubation on ice, cells were transferred to 37 °C for 20 min, washed to remove cell surface-bound, non-internalized proteins and then analyzed by flow cytometry. Results presented are mean values from three experiments ± SEM. The *t*-test was used to assess the statistical significance of measured differences in internalization; * *p* < 0.05, n.s.—not significant.

We have recently demonstrated that galectin-1 and -3 bind FGFR1, affecting receptor activity and cellular transport [18]. To study if galectin-1 and -3 affect KCK-FGF1.E internalization into U2OS-R1 cells, cells were washed with lactose to remove endogenous galectins and treated with purified recombinant galectin-1 and -3 (Figure 2A) prior to flow cytometry measurements. As shown in Figure 2B, supplementation of cells deprived of endogenous galectins with recombinant galectin-1 had no effect on cellular uptake of KCK-FGF1.E. In contrast, supplementation of cells with recombinant galectin-3 partially restored KCK-FGF1E internalization. These data implicate that the galectin network (especially oligomeric galectin-3 extensively implicated in endocytosis) may be involved in the uptake of some CCs targeting FGFR1 [19].

### 2.3. Differential Effects of N-Glycosylation of RTKs on Their Recognition by the Conjugates

**Figure 3:**
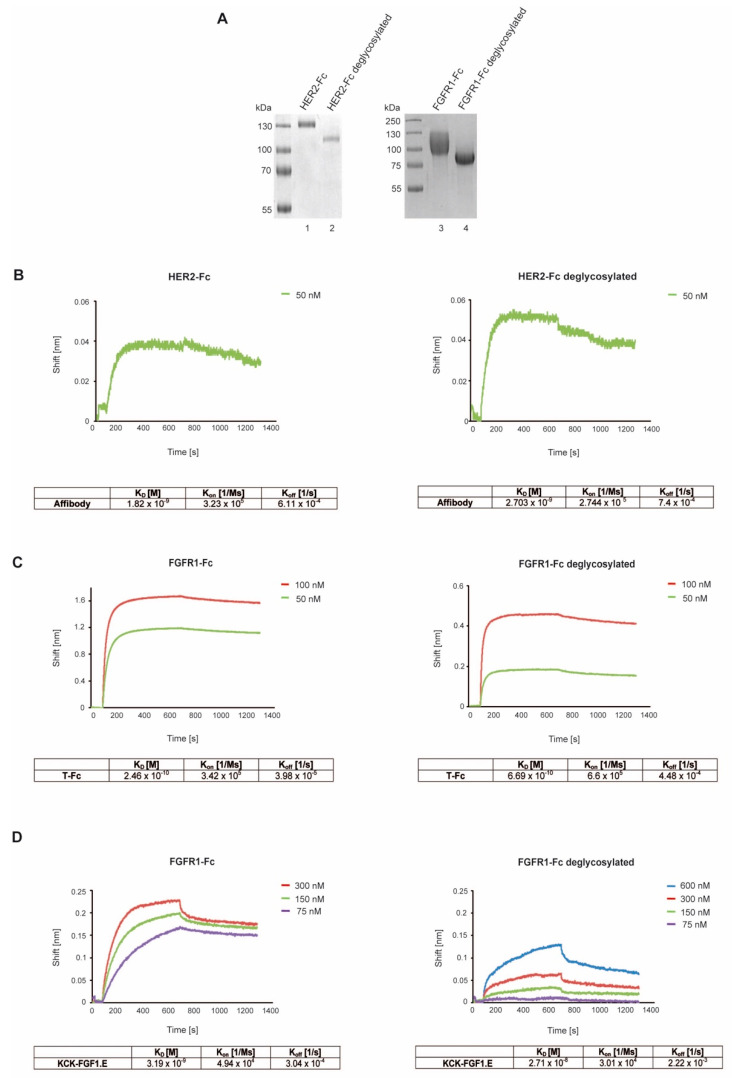
The significance of N-glycosylation for the interaction of CCs with RTKs. (**A**) SDS-PAGE analysis of enzymatic deglycosylation of HER2-Fc and FGFR1-Fc using PNGase F. (**B**–**D**) BLI-determined kinetic parameters of targeting protein interactions with FGFR1-Fc, HER2-Fc and their de-glycosylated variants. HER2-Fc or FGFR1-Fc and deglycosylated receptors were immobilized on BLI sensors and incubated with various concentrations of Affibody_HER2_, T-Fc and KCK-FGF1.E. K_D_, k_on_ and k_off_ were calculated using ForteBio Data Analysis 11.0 software (Pall ForteBio, San Jose, CA, USA).

Reduced internalization of HER2 and FGFR1-specific conjugates following inhibition of cell surface N-glycosylation may be caused by impaired recognition of de-glycosylated receptors by conjugate targeting molecules. Therefore, we measured the kinetic parameters of the interaction between Affibody_HER2_, KCK.FGF1.E and T-Fc, and recombinant wild-type (N-glycosylated) or de-glycosylated HER2 and FGFR1 using biolayer interferometry (BLI). Prior to BLI experiments, the N-glycans of HER2 and FGFR1 were enzymatically removed with PNGase F, as confirmed by accelerated receptor migration in SDS-PAGE (Figure 3A). Virtually the same affinities of Affibody_HER2_ for N-glycosylated and de-glycosylated HER2 were measured, implicating that HER2 glycosylation status does not affect its recognition by Affibody_HER2_ (Figure 3B). Removal of N-linked sugar chains from FGFR1 weakly reduced the interaction of the receptor with T-Fc (Figure 3C). In contrast, de-glycosylation of FGFR1 decreased the affinity of KCK-FGF1.E for FGFR1 almost 10-fold (Figure 3D). This differential dependence of T-Fc and KCK-FGF1.E interaction with FGFR1 on receptor N-glycosylation is likely a result of their distinct binding sites on FGFR1. T-Fc recognizes the N-terminal sequence of the D1 domain of FGFR1 away from the predicted N-glycosylation, whereas KCK-FGF1.E binds to the D2 and D3 domains of FGFR1 containing six predicted N-glycosylation sites [9,16,20].

These data implicate that the significantly reduced cellular uptake of RTK-specific targeting molecules upon N-glycosylation inhibition was not due to inhibition of their binding to de-glycosylated receptors but rather to compromised overall cellular endocytic activity. Furthermore, our data suggest that alteration of N-glycosylation for certain pairs of receptors and targeting molecules may affect the strength and kinetics of their interaction.

### 2.4. Significance of Cell Surface N-Glycosylation for the Potency of CCs Targeting RTKs

**Figure 4:**
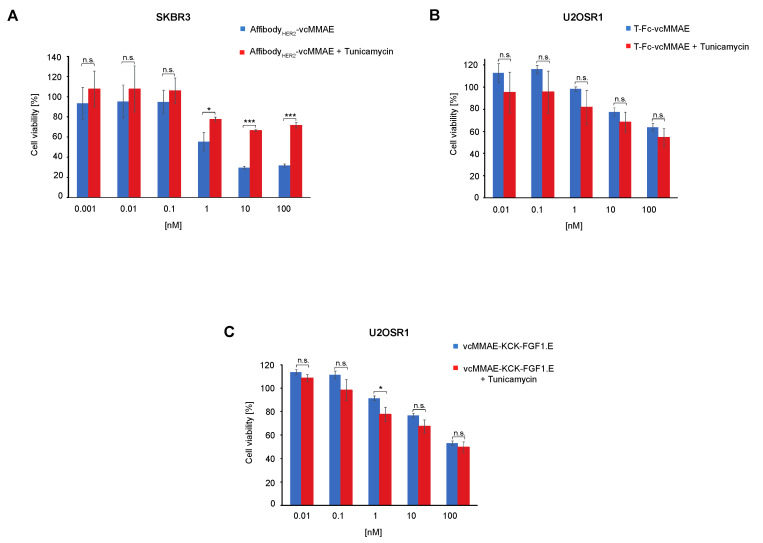
The role of cell surface N-glycosylation in the cytotoxicity of CCs. Cytotoxicity of CCs against FGFR1- or HER2-overproducing cells lacking N-glycosylation. The cytotoxic potential of Affibody_HER2_-vcMMAE (**A**) was measured using SKBR3 cell line, and cytotoxic potential of T-Fc-vcMMAE (**B**) and vcMMAE-KCK-FGFE.1 (**C**) were measured using U2OSR1 cell line. Cells were incubated with tunicamycin 24 h before the experiment and treated with the indicated agents at various concentrations for 96 h, and cell viability was assessed in the Presto Blue assay. Results are mean values from three independent experiments ± SD. Statistical significance: * *p* < 0.05, *** *p* < 0.0001, n.s.—not significant.

Finally, we evaluated whether the significant effect of cell surface N-glycosylation on CC internalization is reflected in their cytotoxic properties. SKBR3 and U2OSR1 cells were pre-treated with tunicamycin prior to supplementation with RTK-specific CCs, and cell viability was assessed using Presto Blue reagent. As shown in Figure 4A, inhibition of N-glycosylation drastically reduced the cytotoxicity of Affibody_HER2_-vcMMAE conjugate for HER2-positive SKBR3 cells.

These data correspond well with the results of the internalization studies (Figure 1D), indicating that the downregulation of cell surface N-glycosylation blocks cell entry and consequently limits the potency of HER2-targeting Affibody_HER2_-vcMMAE. In contrast, pre-treatment of U2OS-R1 cells with tunicamycin had no effect on the cytotoxicity of T-Fc-vcMMAE and vcMMAE-KCK-FGF1.E (Figure 4B,C). The lack of correlation between the efficiency of internalization and the cytotoxic potency of T-Fc-vcMMAE and vcMMAE-KCK-FGF1.E is likely a result of the generally high endocytic potential of FGFR1. This allows intracellular delivery of sufficient drug molecules to induce cell death, even when internalization is partially blocked [21]. HER2, which is considered a low internalizing receptor, is more susceptible to N-glycosylation-dependent modulation of internalization [22]. The effective threshold of Affibody_HER2_-vcMMAE molecules is not reached inside SKBR3 cells when N-glycosylation is blocked, which directly translates into a reduced cytotoxic potential of the HER2-specific conjugate.

## 3. Discussion

Cytotoxic conjugates are sophisticated and highly promising therapeutics for targeted treatment of diverse cancers [1,2,4]. RTKs constitute a large family of cell surface glycoproteins strongly implicated in diverse cancer types and are intensively explored as molecular targets for therapies with CCs [5]. The success of RTK-specific CCs largely depends on the precise recognition of cell surface receptors by CCs and subsequent efficient CC uptake by cancer cells [1,3]. CCs, before reaching the target RTK, have to pass through the dense extracellular matrix that is highly enriched in glycoproteins and glycolipids. These glycoconjugates affect numerous cellular processes, including membrane dynamics and endocytosis and may thus modulate cellular uptake of CCs [17]. Cell surface glycoconjugates closely co-operate with galectins, a group of secreted lectins, in determining the endocytic activity of the cell, and this interplay can affect the efficiency of CC internalization and their toxicity [8]. To date, the impact of cell surface N-glycosylation and galectins in the internalization and toxicity of CCs targeting RTKs has not been studied.

Using CC targeting model RTKs HER2 and FGFR1, we demonstrated that N-glycosylation of cell surface proteins is critical for the efficient uptake of CCs. The inhibition of N-glycosylation largely decreased the internalization of all three tested conjugates. Importantly, we showed that the downregulation of CC endocytosis was not caused by an impaired recognition of RTKs by CCs. We also demonstrated that decreased cellular uptake of CC upon N-glycosylation blockade resulted in a drastic reduction in the toxicity of CC targeting the HER2 receptor. Most likely, the reduced toxicity of the HER2-targeting conjugate is due to reduced endocytosis of glycosylation-deficient HER2. Alternatively, the interactome and organization of HER2 in lipid microdomains might be altered upon N-glycosylation blockade, affecting HER2 recognition by the conjugate. Modification of cell surface glycosylation profile in cancer is a well-described phenomenon that contributes to oncogenesis at many different levels. Extensive remodeling of N-glycans on the surface of cancer cells may alter the endocytosis of CCs, representing a novel defense mechanism against CCs [23]. This mechanism would be especially effective against CCs targeting slowly internalizing cell surface receptors, such as HER2 [22].

Receptor-mediated cellular uptake of CCs might occur via several distinct endocytic routes, and pathway choice is largely determined by the receptor type [23]. Galectins are well-known endocytic modulators that can either activate endocytosis by inducing galectin-specific clathrin-independent endocytosis or block internalization of cell surface receptors by their extensive clustering on the cell surface [8]. Since several RTKs, including HER2 and FGFRs, directly or indirectly interact with galectins, we decided to determine the role of galectins in the internalization of CCs targeting model RTKs [9,18]. We found that removal of endogenous galectins with lactose partially blocked the cellular uptake of FGFR1-specific KCK-FGF1.E. We were able to restore the endocytosis of KCK-FGF1.E with recombinant galectin-3, a chimeric galectin strongly involved in membrane dynamics, which implicates that a galectin network of defined composition might modulate receptor-mediated endocytosis of specific CCs.

Most RTKs, including HER2 and FGFR1, are N-glycosylated at several positions [9]. While we observed that removal of N-linked sugar chains from HER2 and FGFR1 had virtually no effect on their interaction with Affibody_HER2_ and T-Fc, respectively, it significantly blocked FGFR1 interaction with KCK-FGF1.E. This effect is likely caused by the fact that an FGF binding site formed by D2 and D3 domains of FGFR1 is particularly rich in N-glycosylation sites. These data suggest that N-glycosylation of cancer-specific receptors may in some cases alter receptor recognition by targeting molecules in CCs and should be taken into consideration during CC engineering.

Altogether, our data implicate that cell surface N-glycosylation and the interplay between plasma membrane glycoconjugates and the galectin network is important for the efficient uptake and potency of CC conjugates targeting cancer-relevant RTKs.

## 4. Materials and Methods

### 4.1. Antibodies and Reagents

The primary antibodies directed against FGFR1 (#9740) were from Cell Signaling (Danvers, MA, USA), anti-HER2 primary antibodies (sc-33684, sc-8036) were from Santa Cruz Biotechnology (Dallas, TX, USA), and anti-tubulin primary antibodies (#T6557) were from Sigma-Aldrich (St. Louis, MO, USA). Secondary antibodies coupled to HRP were from Jackson Immuno-Research Laboratories (Cambridge, UK). Tunicamycin was from Santa Cruz Biotechnology. DyLight™ 550 NHS Ester used for fluorescent protein labeling was from Thermo Fisher Scientific (Waltham, MS, USA).

### 4.2. Recombinant Proteins

Fully glycosylated extracellular domain of FGFR1 fused to the Fc fragment of human IgG1: FGFR1 IIIc (FGFR1-Fc) was produced as described previously by our group [24]. Recombinant human HER2-Fc chimera protein was obtained from biotechne (Minneapolis, MN, USA). T-Fc was expressed, purified and conjugated with vcMMAE according to [11]. The expression, purification and conjugation of Affibody_HER2_ and KCK-FGF1.E were performed as described in [20]. Fluorescent labeling of T-Fc, KCK-FGF1.E and Affibody_HER2_ was performed as described in manufacturer’s instructions (Thermo Fisher Scientific). Recombinant galectin-1 and galectin-3 were produced as described in [18].

### 4.3. Cell Culture

Human breast adenocarcinoma cell line (SKBR3) was obtained from American Type Culture Collection (ATCC) (Manassas, VA, USA). U2OS cells stably expressing FGFR1 (U2OSR1) were obtained by transfection of U2OS cells with expression plasmid encoding FGFR1 as described in [11]. Cells were cultured according to [20].

### 4.4. BLI Measurements

Kinetic parameters of the interaction of the analyzed proteins with HER2 and FGFR1 were determined by bio-layer interferometry (BLI) using ForteBio Octet K2 (Pall ForteBio, San Jose, CA, USA). Enzymatic de-glycosylation of FGFR1-Fc and HER2-Fc was performed using PNGase F for 4 h, according to the manufacturer’s recommendations (New England Biolabs, Ipswich, MA, USA). Measurements were performed analogously to the experiments presented in [20].

### 4.5. Flow Cytometry

U2OSR1 and SKBR3 cells were treated with 0.5 μg/mL tunicamycin for 24 h or with 50 mM lactose for 15 min before the experiment. The internalization of fluorescently labeled T-Fc (30 nM), Affibody_HER2_ (30 nM) and KCK-FGF1.E (30 nM) under various conditions was analyzed according to^12^. To investigate the effect of galectins on the internalization of the analyzed proteins, galectin-1 (5 μg/mL) and galectin-3 (5 μg/mL) were added to cells with the tested proteins prior to flow cytometry measurements. Cells were analyzed using a NovoCyte 2060R Flow Cytometer and NovoExpress software (ACEA Biosciences, San Diego, CA, USA).

### 4.6. Cytotoxicity Assay

U2OSR1 and SKBR3 cells were treated with 0.5 μg/mL tunicamycin for 24 h. The cytotoxicity of T-Fc-vcMMAE, Affibody_HER2_-vcMMAE and KCK-FGF1.E-vcMMAE was then analyzed as in [13,20].

## Data Availability

Not applicable.
